# Predicted but not destined: considerations for obesity-related cardiovascular risk projection

**DOI:** 10.1016/j.lanwpc.2023.100828

**Published:** 2023-06-19

**Authors:** Tzung-Dau Wang, Chung-Lieh Hung, Hung-I Yeh

**Affiliations:** a Cardiovascular Center and Divisions of Cardiology and Hospital Medicine, National Taiwan University Hospital, Taipei City, Taiwan; b Division of Cardiology, Department of Internal Medicine, MacKay Memorial Hospital, Taipei City, Taiwan; c Institute of Biomedical Sciences, MacKay Medical College, New Taipei City, Taiwan

The main difference between the top 2 killers, cardiovascular diseases and malignancies, is that cardiovascular diseases are mostly preventable or, more accurately, explainable, whereas most malignancies are not. More than two decades ago, the case–control INTERHEART study clearly demonstrated that nine risk factors, including dyslipidemia, hypertension, diabetes mellitus, abdominal obesity, smoking, dietary pattern, alcohol consumption, psychosocial factors, and regular physical activity, accounted for more than 90% of the risk of myocardial infarction in both sexes and at all ages among 15,152 cases of acute myocardial infarction (AMI) and 14,820 controls from 52 countries.^1^ Likewise, the American Heart Association advocates metrics summarized as Life's Essential 8, including diet, physical activity, nicotine exposure, sleep health, body mass index, blood lipids, blood glucose, and blood pressure, to assess overall cardiovascular health and achieve health promotion and preservation.^2^

Given the modifiable nature of atherosclerotic cardiovascular diseases, it is essential to estimate the evolution of the impact of recognized risk factors for proactive healthcare policy planning. In the latest issue of *The Lancet Regional Health Western Pacific*, Chew et al.^3^ used nationwide data from the Singapore Myocardial Infarction Registry from 2007 to 2018 to estimate the incidence and mortality of AMI and five risk factors (diabetes mellitus, hypertension, hyperlipidemia, overweight/obesity, and active/previous smoking) among patients between 2025 and 2050. In the Singapore Myocardial Infarction Registry, patients with AMI are identified by screening for elevated troponin levels above the 99th percentile of each laboratory, confirming its comprehensive national coverage. Although all registered patients with AMI underwent central verification by trained personnel, all types of myocardial infarction, rather than simply type 1,^4^ were included in the registry. By using both Poisson and linear regression models, the authors predicted that, from 2025 to 2050, the incidence of AMI would rise by 194.4% from 482 to 1418 per 100,000 population. The largest increase in the incidence of risk factors in patients with AMI is projected to occur in the overweight/obesity category, defined by body mass index (880% increase, from 384 to 3764 per 100,000 population). AMI-related mortality is projected to decrease by 58.6%, from 19 to 8 per 100,000 population. AMI-related mortality is projected to increase by 193.4% in overweight/obese patients (from 12 to 34 per 100,000 population), whereas mortality is predicted to decrease in patients with AMI and each of the remaining four risk factors. The authors also projected that Indian and Malay populations bear a greater burden of overweight/obesity incidence and mortality in patients with AMI than the Chinese population.

This projection analysis highlights that, together with the continuing rise in the incidence of AMI in the coming decades, overweight/obesity is set to overtake hypertension, diabetes, and hyperlipidemia as the leading risk factor for AMI by 2050 in Singapore. This information adds to the overwhelming evidence that the obesity pandemic threatens humanity in an insidious and relentless manner in the Western Pacific region. However, methodological issues are worth mentioning, and caution should be exercised when interpreting the results presented. First, since actual numbers rather than relative percentages of risk factors in patients with AMI were used to generate the projection, the resultant absolute number of patients with AMI and overweight/obesity appears to surpass the overall number of patients with AMI since 2030,^3^ perhaps partly due to the overestimation of the projections of obesity.^3^ Second, overweight/obesity was defined as a body mass index of ≥23 kg/m^2^, the cut-off value used for Asian populations. It would be of interest to know whether obesity alone, as defined as body mass index of ≥27.5 kg/m^2^ for Asian populations,^5^ could still be shown as the leading risk factor. Nevertheless, the importance of weight control in patients with AMI or those who are overweight/obese should not be overestimated. Third, as recognized by the researchers, all the projections are based on the assumption that the trends observed in the analysis of past data will remain consistent moving forward, which may inherently overestimate the recent progress in the management of AMI (thus, the extremely low rate of AMI-related mortality projected in 2050, 0.6% [8/1418]) and the uncontrolled obesity pandemic.

Despite the projections related to obesity in the present study, the lack of obesity phenotype measures such as central obesity or body composition may limit the precise interpretation of the pathophysiological interplay between excessive adiposity and cardiovascular events. Although only body mass index was used to define overweight/obesity in this study, we and other investigators have demonstrated that body mass index, irrespective of waist circumference and waist-to-hip ratio, is independently associated with metabolic syndrome in Asian populations.^6^^,^^7^ Studies on the use of novel treatments for overweight/obesity (such as semaglutide and tirzepatide) for the effective amelioration of cardiovascular events are under investigation,^8^, ^9^, ^10^ which may further revolutionize the landscape of primary and secondary prevention of cardiovascular diseases.

Given the global epidemic of obesity with the Western Pacific region at the forefront, all stakeholders, including patients, healthcare professionals, societies, and governments, should work wholeheartedly to combat this overweight/obesity pandemic and ensure that it does not continue on its projected path.

## Contributors

Conceptualization, C.L.Hung; validation, C.L.Hung, T.D. Wang and H.I Yeh; writing—original draft preparation, T.D. Wang; writing—review and editing, C.L.Hung and H.I Yeh. All authors have read and agreed to the published version of the manuscript.

## Declaration of interests

We declare no competing interests.
